# Unveiling the relative efficacy, safety and tolerability of prophylactic medications for migraine: pairwise and network-meta analysis

**DOI:** 10.1186/s10194-017-0720-7

**Published:** 2017-02-20

**Authors:** Aijie He, Dehua Song, Lei Zhang, Chen Li

**Affiliations:** 1 Department of Neurosurgery, the Affiliated Yantai Yuhuangding Hospital of Qingdao University, 264000 Yantai, Shandong China; 2 Department of Radiotherapy, the Affiliated Yantai Yuhuangding Hospital of Qingdao University, 264000 Yantai, Shandong China; 3 Department of Pharmacy, Yantai Hospital of Traditional Chinese Medicine, 264000 Yantai, Shandong China; 4 Department of Anesthesia, Yantai Hospital of Traditional Chinese Medicine, No. 39 Xingfu Road, Zhifu Disctrict, 264000 Yantai, Shandong China

**Keywords:** Migraine, Efficacy, Safety, Tolerability, Network meta-analysis

## Abstract

**Background:**

A large number patients struggle with migraine which is classified as a chronic disorder. The relative efficacy, safety and tolerability of prophylactic medications for migraine play a key role in managing this disease.

**Methods:**

We conducted an extensive literature search for popular prophylactic medications that are used for migraine patients. Pairwise meta-analysis and network meta-analysis (NMA) were carried out sequentially for determining the relative efficacy, safety and tolerability of prophylactic medications. Summary effect for migraine headache days, headache frequency, at least 50% reduction in headache attacks, all-adverse events, nausea, somnolence, dizziness, withdrawal and withdrawal due to adverse events were produced by synthesizing both direct and indirect evidence.

**Results:**

Patients with three interventions exhibited significantly less average migraine headache days compared with those treated by placebo (topiramate, propranolol, divalproex). Moreover, topiramate and valproate exhibited a significantly increased likelihood of at least 50% reduction in migraine headache attacks compared to placebo. Patients with topiramate and propranolol also exhibited significantly reduced headache frequency compared to those with placebo. On the other hand, patients with divalproex exhibited significantly higher risk of nausea compared to those with placebo, topiramate, propranolol, gabapentin and amitriptyline. Finally, divalproex was associated with an increased risk of withdrawal compared to placebo and propranolol.

**Conclusions:**

Topiramate, propranolol and divalproex may be more efficacious than other prophylactic medications. Besides, the safety and tolerability of divalproex should be further verified by future studies.

**Electronic supplementary material:**

The online version of this article (doi:10.1186/s10194-017-0720-7) contains supplementary material, which is available to authorized users.

## Background

Migraine is a chronic neurological disorder with high prevalence. Females appeared to have a higher morbidity of migraine than males in developed countries [1]. Although a relatively small number of migraine cases were reported in Asia, the morbidity of migraine attack in this region can reach up to 9.3% [2]. Throbbing headache is usually accompanied with migraine, resulting in both poor productivity and unstable emotional state [3, 4]. Migraine patients are often managed by medications which are convenient and efficient. However, side effects such as nausea and dizziness resulted from these medications have been observed in patients who exhibited poor level of tolerance [5].

Two types of medications have been introduced to patients: abortive and preventative medications [6]. The above two types of medications differ considerably in their mechanisms: abortive treatments attenuate symptoms arise from acute migraine attacks whereas preventative medications specifically aim at reducing attack severity and frequency. Although several prophylactic medications have been developed for migraine patients, no consensus has been reached with respect to their relative efficacy, safety and tolerability [7]. Furthermore, some medications appear to provide inadequate relief since they are not effective to all migraine patients [8]. As a result, some meta-analysis has been designed to compare the relative efficacy between different medications and some conclusions have been obtained in the current literature. For instance, patients treated by sodium valproate were associated with a lower risk of headache compared to the control group [9]. Furthermore, triptans and non-triptans appear to provide patients with different levels of relief [10].

Nevertheless, the current literature does not contain adequate studies that are able to identify the most preferable prophylactic medication for migraine patients and there is an increasing demand for discriminating the available medications with respect to their efficacy, safety and tolerability. For this purpose, we compared several preventative medications for migraine patients by using the approach of network meta-analysis (NMA) and we expect this approach can provide more insights for the selection of prophylactic medications.

## Methods

### Search strategy

**Fig. 1:**
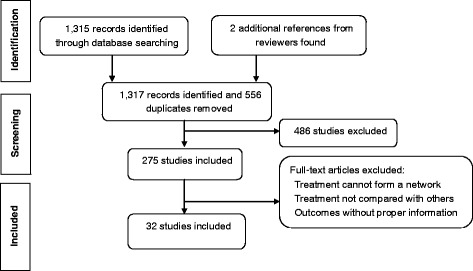
Flow chart of literature identification, search and inclusion

The medical literature for relevant studies in PubMed and EMBASE were systematically searched using electronic search strategies, and 1315 records were identified through searching the following key words, for example “migraine”, “topiramate”, “propranolol”, “gabapentin”, “amitriptyline”, “divalproex” and “valproate”. Two additional references were obtained from reviewers. As flow chart Fig. 1 illustrates, 556 duplicated records were identified and removed. Another 486 irrelevant studies were excluded from the remaining 761 records and a final 32 studies were subject to full-text review (Table 1).

**Table 1:** Studies identified for the NMA with interventions and outcomes evaluated

Author, Year	Center	Design	Blind	Mechanism of action	Intervention	Size	Male (%)	Follow-up	Age	Clinical outcomes
Silberstein et al., 2013 [42]	Multi	RCT	Double	Anticonvulsants	Gabapentin vs. Placebo	523	21	20w	39.4	①③④⑤⑥⑦⑧⑨
Afshari et al., 2012 [41]	Mono	RCT	Double	Anticonvulsants	Topiramate vs. Valproate	56	21	12w	32.1	②④⑤⑥⑧⑨
Lipton et al., 2011 [40]	Multi	RCT	Double	Anticonvulsants	Topiramate vs. Placebo	330	13	26w	39.6	①④⑤⑥⑦⑧⑨
Holroyd et al., 2010 [39]	Multi	RCT	Double	β blocker	Propranolol vs. Placebo	106	21	16m	38.2	①⑧⑨
Dodick et al., 2009 [38]	Multi	RCT	Double	Anticonvulsants	Topiramate vs. Amitriptyline	346	15	26w	38.8	①④⑤⑥⑦⑧⑨
Ashtari et al., 2008 [37]	Mono	RCT	Double	Anticonvulsants	Topiramate vs. Propranolol	62	21	8w	30.5	②⑥⑦⑧⑨
Silberstein et al., 2007 [36]	Multi	RCT	Double	Anticonvulsants	Topiramate vs. Placebo	306	15	126d	38.2	①④⑤⑥⑦⑧⑨
Gupta et al., 2007 [35]	Mono	Crossover	Double	Anticonvulsants	Topiramate vs. Placebo	60	22	20w	29.4	①④⑧⑨
Diener et al., 2007 [33, 34]	Multi	RCT	Double	Anticonvulsants	Topiramate vs. Placebo	59	26	16w	46.2	①④⑤⑥⑦⑧
Diener et al., 2007 [33, 34]	Multi	RCT	Double	Anticonvulsants	Topiramate vs. Placebo	514	13	6m	39.8	①④⑤⑦⑧
Tommaso et al., 2007 [32]	Mono	RCT	Double	Anticonvulsants	Topiramate vs. Placebo	30	22	1d	37.9	⑨
Silberstein et al., 2006 [31]	Multi	RCT	Double	Anticonvulsants	Topiramate vs. Placebo	211	14	12w	40.5	④
Shaygannejad et al., 2006 [30]	Mono	Crossover	Double	Anticonvulsants	Topiramate vs. Valproate	64	57	24w	34.1	②⑥
Brandes et al., 2006 [29]	Multi	RCT	Double	Anticonvulsants	Topiramate vs. Placebo	468	13	13m	38.8	②⑧⑨
Silberstein et al., 2004 [28]	Multi	RCT	Double	Anticonvulsants	Topiramate vs. Placebo	469	11	26w	40.4	①②③⑤⑥⑧⑨
Mei et al., 2004 [27]	Mono	RCT	Double	Anticonvulsants	Topiramate vs. Placebo	115	46	16w	39.2	②③④⑥⑧⑨
Diener et al., 2004 [26]	Multi	RCT	Double	Anticonvulsants	Topiramate vs. Propranolol	568	20	1y	41.0	①②④⑤⑥⑧⑨
Brandes et al., 2004 [25]	Multi	RCT	Double	Anticonvulsants	Topiramate vs. Placebo	483	13	26w	38.8	⑤⑧⑨
Freitag et al., 2002 [24]	Multi	RCT	Double	Anticonvulsants	Divalproex vs. Placebo	237	21	12w	40.5	①②③④⑤⑥⑧⑨
Storey et al., 2001 [23]	Mono	RCT	Double	Anticonvulsants	Topiramate vs. Placebo	40	25	12w	38.2	②③⑧⑨
Mathew et al., 2001 [22]	Multi	RCT	Double	Anticonvulsants	Gabapentin vs. Placebo	143	17	16w	39.4	②③④⑥⑦⑧⑨
Klapper, 1997 [21]	Multi	RCT	Single	Anticonvulsants	Divalproex vs. Placebo	176	12	12w	40.5	③④⑤⑥⑧⑨
Kaniecki, 1997 [20]	Mono	RCT	Single	Anticonvulsants	Divalproex vs. Propranolol	37	19	12w	40.1	④⑤⑦⑨
Diener et al, 1996 [19]	Multi	RCT	Double	β blocker	Propranolol vs. Placebo	133	22	12w	39.0	③⑧⑨
Bendtsen et al., 1996 [18]	Mono	Crossover	Double	Antidepressive	Amitriptyline vs. Placebo	40	38	32w	40.0	④⑤⑦⑨
Mathew et al., 1995 [17]	Multi	RCT	Double	Anticonvulsants	Divalproex vs. Placebo	107	20	12w	46.0	①③⑤⑥⑧⑨
Hering and Kuritzky, 1992 [16]	Mono	Crossover	Double	Anticonvulsants	Valproate vs. Placebo	32	21	16w	34.0	⑤⑦⑨
Pradalier et al., 1989 [15]	Multi	RCT	Double	β blocker	Propranolol vs. Placebo	74	24	16w	37.4	②⑤⑦⑧⑨
Mikkelsen et al., 1986 [14]	Mono	Crossover	Double	β blocker	Propranolol vs. Placebo	31	16	12w	38.0	④⑧⑨
Sadeghian andMotiei-Langroudi, 2015 [45]	Mono	RCT	Double	Anticonvulsants	Valproate vs. Placebo	58	27	6m	35.3	③④⑨
Sarchielli et al., 2014 [44]	Multi	RCT	Double	Anticonvulsants	Valproate vs. Placebo	88	21	6m	42.0	④⑤⑦⑧⑨
Nofal et al., 2014 [43]	Moni	RCT	Double	Anticonvulsants	Gabapentin vs. Placebo	86	0	4d	30.9	⑤⑦

Outcomes: ① monthly migraine headache days; ② headache frequency; ③ ≥50% reduction in migraine headache attacks; ④ all-adverse events; ⑤ nausea; ⑥ somnolence; ⑦ dizziness; ⑧ withdrawal; ⑨ withdrawal due to adverse events

### Exclusion criteria

Articles were excluded in our study according to the following criteria: (1) the diagnose of migraine was not firmly confirmed in the study; (2) contain treatments that cannot form a closed network; (3) have no comparisons between different treatments; (4) contain outcomes without proper information; (5) does not have any relevant clinical outcomes or treatments; (6) studies without blinding procedures or studies with sample size less than 30; (7) non-randomized clinical trials such as reviews. A study was not considered to be eligible if any of the above criteria was fulfilled.

### Outcome measures, data extraction and comparator network formation

**Fig. 2:**
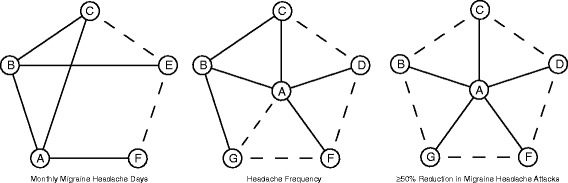
Network plots of eligible comparisons of migraine intervention (monthly migraine headache days; headache frequency; ≥50% reduction in migraine headache attacks). *A*: Placebo; *B*: Topiramate; *C*: Propranolol; *D*: Gabapentin; *E*: Amitriptyline; *F*: Divalproex; *G*: Valproate. Direct comparisons were connected by solid lines whereas indirect comparisons were connected by dashed lines

**Fig. 3:**
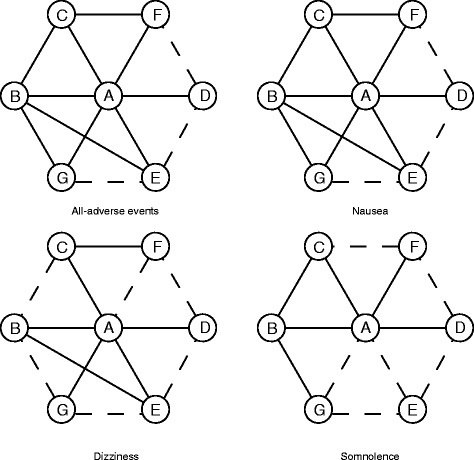
Network plots of eligible comparisons for adverse events (all adverse events; nausea; somnolence; dizziness) *A*: Placebo; *B*: Topiramate; *C*: Propranolol; *D*: Gabapentin; *E*: Amitriptyline; *F*: Divalproex; *G*: Valproate. Direct comparisons were connected by solid lines whereas indirect comparisons were connected by dashed lines

**Fig. 4:**
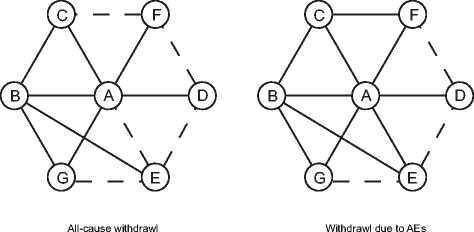
Network plots of eligible comparisons for discontinued cases (all-cause withdrawal; withdrawal due to AEs). A: Placebo; B: Topiramate; C: Propranolol; D: Gabapentin; E: Amitriptyline; F: Divalproex; G: Valproate. Direct comparisons were connected by solid lines whereas indirect comparisons were connected by dashed lines

We selected several clinical outcomes in order to measure the relative efficacy, safety and tolerability of prophylactic migraine medications: monthly migraine headache days, headache frequency, the percentages of patients with at least 50% reductions in migraine attacks (efficacy), the number of patients with all adverse events such as nausea, somnolence or dizziness (safety) and the number of patients who withdrew from studies (tolerability). The following data were extracted from eligible studies and shown in Table 1, including country of study, sample size, histology and clinical outcomes. The corresponding data were extracted into a database after two independent investigators reviewed the manuscripts of all the studies. A Jaded scale table was produced for the purpose of study quality assessment (Additional file 1: Table S1). After data extraction was performed for each study, a network plot with respect to each clinical outcome was produced for demonstrating direct and indirect comparisons (Figs. 2, 3 and 4).

### Statistical analysis

We implemented a two-step approach in our system review and evidence synthesis. Firstly, a conventional pairwise meta-analysis was carried out in order to pool all direct evidence in the current literature. For continuous outcomes such as monthly migraine headache days and headache frequency, raw mean differences (MD) between two groups were synthesized and a summary effect was produced based on raw mean differences, sample size and sample standard deviation. We selected raw mean differences for evidence synthesis since all eligible studies reported the above continuous outcomes in the same scale. On the other hand, the statistic of odds ratio (OR) was pooled from each eligible study and a summary OR was produced for each binary outcome. The pairwise meta-analysis was implemented based on the random-effects model because we did not have full knowledge of study participants or implementation for each eligible study [11].

The second step in our study involves conducting a NMA by synthesize both direct and indirect evidence. Similar to the pairwise meta-analysis, summary mean differences and summary ORs were produced using the Bayesian framework and the Markov Chain Monte Carlo (MCMC) sampling technique. The corresponding ranking of each intervention was obtained by using the surface under the cumulative ranking curve (SUCRA). If an intervention exhibited a higher SUCRA value compared to other interventions, then it is potentially more preferable than others with respect to an endpoint. After that, the assumption of consistency between direct and indirect evidence was assessed by using the node-splitting method whereas publication bias was visually inspected by using funnel plots [12, 13].

## Results

### Description of included studies

The prescribed searching strategy and exclusion criteria enabled us to identify and include 32 studies with a total number of 6052 subjects (Table 1) [14–45]. All included studies were carried out by using single or double blinding procedures. These studies were carried out between 1986 and 2015 with a maximum following-up duration of 1 year. The majority of the included studies belonged to typical randomized controlled trials (RCTs), however, we identified and included five crossover RCTs in which participants were randomized to receive a sequence of interventions.

### Pairwise comparison using conventional meta-analysis

A total of ten direct comparisons with respect to each endpoint were produced by using pairwise meta-analysis (Table 2). Patients with topiramate exhibited significantly less average headache days, less headache frequency, a higher likelihood of at least 50% reduction compared to those with placebo (migraine headache days: −0.28, 95% CI = −0.53 to −0.03; headache frequency: −0.31, 95% CI = −0.45 to −0.17; ≥ 50% reduction: OR = 2.33, 95% CI = 1.58–3.42). However, patients with topiramate appeared to have significantly higher risk of all-adverse events and withdrawal due to adverse events compared to those with placebo (all-adverse events: OR = 1.35, 95% CI = 1.06–1.73, withdrawal due to adverse events: OR = 2.08, 95% CI = 1.56–2.78). Patients with propranolol exhibited a significantly less average headache days but higher risk of all-adverse events, somnolence and withdrawal due to adverse events compared to those with placebo (migraine headache days: −0.29, 95% CI = −0.49 to −0.09; all-adverse events: OR = 2.02, 95% CI = 1.05–4.08, somnolence: OR = 4.33, 95% CI = 1.21 to 15.53, withdrawal due to adverse events: OR = 1.87, 95% CI = 1.09 to 3.09). Although there is no significant differences in the average migraine days, headache frequency or the likelihood of at least 50% reduction in headache attacks between patients with gabapentin and those with placebo, gabapentin appeared to be associated with an increased risk of somnolence and dizziness (somnolence: OR = 2.23, 95% CI = 1.11 to 4.46; dizziness: OR = 3.13, 95% CI = 1.73 to 5.56). Patients treated with amitriptyline or divalproex exhibited a reduced headache days or headache frequency as well as a better performance in at least 50% reduction in headache attacks compared to those with placebo (amitriptyline: headache frequency: −0.36, 95% CI = −0.62 to −0.10; ≥ 50% reduction: OR = 1.81, 95% CI = 1.03–3.20; divalproex: migraine headache days: −0.40, 95% CI = −0.61 to −0.18; ≥ 50% reduction: OR = 4.27, 95% CI = 1.30–13.99), however, this was offset by an increased risk of all-adverse events or nausea (amitriptyline: all-adverse events: OR = 2.20, 95% CI = 1.04–4.66; divalproex: nausea: OR = 2.23, 95% CI = 1.21–4.10). Besides that, we were not able to identify any significant results between direct comparisons produced by conventional meta-analysis. Besides, propranolol was safer comparing to topiramate (all-adverse events: OR = 0.57, 95% CI = 0.36–0.90; withdrawal: OR = 0.66, 95% CI = 0.44–0.99; withdrawal due to adverse events: OR = 0.58, 95% CI = 0.37–0.91).

**Table 2:** Relative treatment efficacy, safety and tolerability produced by pairwise meta-analysis

Comparison	Migraine headache days	Headache frequency	≥50% Reduction	All-adverse events	Nausea	Somnolence	Dizziness	Withdrawal	Withdrawal due to AEs
Placebo vs Topiramate	**−0.28 (−0.53, −0.03)**	**−0.31 (−0.45, −0.17)**	**2.33 (1.58, 3.42)**	**1.35 (1.06, 1.73)**	1.31 (0.97, 1.76)	1.38 (0.70, 2.74)	1.07 (0.54, 2.13)	1.05 (0.91, 1.21)	**2.08 (1.56, 2.78)**
Placebo vs Propranolol	**−0.29 (−0.49, −0.09)**	−1.17 (−2.89, 0.55)	1.37 (0.69, 2.70)	**2.02 (1.05, 4.08)**	1.64 (0.78, 3.47)	**4.33 (1.21, 15.53)**	1.27 (0.20, 8.08)	1.07 (0.76, 1.51)	**1.87 (1.09, 3.19)**
Placebo vs Gabapentin	−0.09 (−0.29, 0.10)	−0.34 (−0.69, 0.01)	1.36 (0.63, 2.95)	1.15 (0.87, 1.51)	0.92 (0.52, 1.64)	**2.23 (1.11, 4.46)**	**3.13 (1.73, 5.66)**	1.21 (0.82, 1.77)	1.57 (0.86, 2.88)
Placebo vs Amitriptyline	-	**−0.36 (−0.62, −0.10)**	**1.81 (1.03, 3.20)**	**2.20 (1.04, 4.66)**	0.33 (0.03, 3.34)	-	1.75 (0.47, 6.45)	-	2.00 (0.17, 22.93)
Placebo vs Divalproex	**−0.40 (−0.61, −0.18)**	-	**4.27 (1.30, 13.99)**	0.98 (0.71, 1.34)	**2.23 (1.21, 4.10)**	1.92 (0.32, 11.63)	-	1.61 (0.92, 2.82)	1.67 (0.70, 3.98)
Placebo vs Valproate	-	-	-	-	3.00 (0.59, 15.37)	-	2.00 (0.36, 11.26)	0.88 (0.29, 2.62)	0.97 (0.26, 3.56)
Topiramate vs Propranolol	−0.12 (−0.32, 0.08)	0.18 (−0.45, 0.81)	-	**0.57 (0.36, 0.90)**	0.81 (0.45, 1.45)	1.42 (0.68, 2.99)	-	**0.66 (0.44, 0.99)**	**0.58 (0.37, 0.91)**
Topiramate vs Amitriptyline	0.01 (−0.20, 0.22)	-	-	1.03 (0.76, 1.41)	0.70 (0.33, 1.49)	1.50 (0.82, 2.72)	1.26 (0.61, 2.57)	1.02 (0.70, 1.50)	1.14 (0.69, 1.88)
Topiramate vs Valproate	-	-	-	1.22 (0.54, 2.76)	1.00 (0.29, 3.48)	1.30 (0.44, 3.84)	-	0.67 (0.24, 1.88)	2000 (0.58, 18.16)
Propranolol vs Divalproex	-	-	-	1.36 (0.58, 3.16)	3.50 (0.67, 18.15)	-	1.00 (0.19, 5.33)	-	4.00 (0.42, 37.78)

Boldface means significance

### Including both direct and indirect evidence in the NMA

**Fig. 5:**
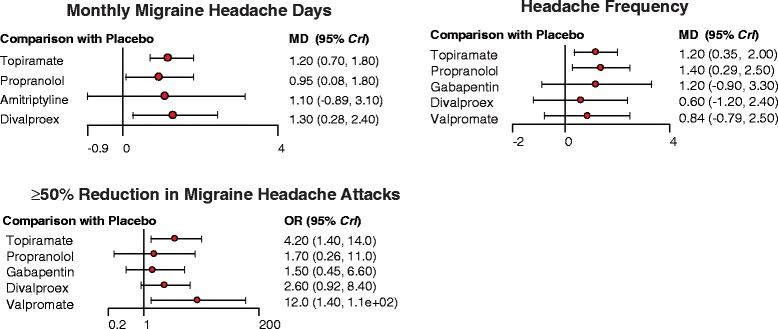
Forest plots of summary effects (NMA) with respect to monthly migraine headache days, headache frequency and at least 50% reduction in migraine attacks

Results produced by NMA are displayed in Table 3 which determined the relative efficacy, safety and tolerability of prophylactic migraine interventions by using both direct and indirect evidence. Patients with three interventions exhibited significantly less average migraine headache days compared with those treated by placebo (topiramate: −1.20, 95% CrI = −1.83 to −0.70; propranolol: −0.98, 95% CrI = −1.86 to −0.07; divalproex: −1.28, 95% CrI = −2.44 to −0.27; Table 3, Fig. 5). Moreover, patients with topiramate and valproate exhibited a significantly increased likelihood of at least 50% reduction in migraine headache attacks compared to those with placebo (topiramate: OR = 4.28, 95% CrI = 1.35 to 14.70; valproate: 11.38, 95% CrI = 1.31 to 111.11; Table 3, Fig. 5). Patients with topiramate or propranolol also exhibited significantly reduced headache frequency compared to those with placebo (topiramate: −1.17, 95% CrI = −1.98 to −0.35; propranolol: −1.37, 95% CrI = −2.49 to −0.29; Table 3, Fig. 5).

**Table 3:** Relative efficacy, safety and tolerability of migraine interventions produced by NMA

Migraine Headache Days	**Placebo**	**Placebo**	1.20 (0.70, 1.83)	0.98 (0.07, 1.86)	-	1.09 (−0.89, 3.13)	1.28 (0.27, 2.44)	-
	**Topiramate**	**−1.20 (−1.83, −0.70)**	**Topiramate**	−0.22 (−1.30, 0.67)	-	−0.10 (−2.03, 1.81)	0.09 (−1.16, 1.31)	-
	**Propranolol**	**−0.98 (−1.86, −0.07)**	0.22 (−0.67, 1.30)	**Propranolol**	-	0.13 (−2.02, 2.33)	0.31 (−1.05, 1.83)	-
	**Gabapentin**	-	-	-	**Gabapentin**	-	-	-
	**Amitriptyline**	−1.09 (−3.13, 0.89)	0.10 (−1.81, 2.03)	−0.13 (−2.33, 2.02)	-	**Amitriptyline**	0.21 (−2.08, 2.53)	-
	**Divalproex**	**−1.28 (−2.44, −0.27)**	−0.09 (−1.31, 1.16)	−0.31 (−1.83, 1.05)	-	−0.21 (−2.53, 2.08)	**Divalproex**	-
	**Valproate**	-	-	-	-	-	-	**Valproate**
Headache Frequency								
≥50% Reduction in Migraine Headache Attacks	**Placebo**	**Placebo**	**1.17 (0.35, 1.98)**	**1.37 (0.29, 2.49)**	1.20 (−0.87, 3.28)	-	0.60 (−1.18, 2.42)	0.84 (−0.81, 2.48)
	**Topiramate**	**4.28 (1.35, 14.70)**	**Topiramate**	0.21 (−0.88, 1.33)	0.05 (−2.20, 2.28)	-	−0.56 (−2.53, 1.39)	−0.32 (−1.76, 1.10)
	**Propranolol**	1.65 (0.25, 11.29)	0.38 (0.04, 3.70)	**Propranolol**	−0.17 (−2.45, 2.15)	-	−0.76 (−2.90, 1.32)	−0.53 (−2.33, 1.23)
	**Gabapentin**	1.59 (0.41, 6.93)	0.37 (0.06, 2.43)	0.96 (0.10, 10.96)	**Gabapentin**	-	−0.61 (−3.39, 2.12)	−0.36 (−3.01, 2.38)
	**Amitriptyline**	-	-	-	-	**Amitriptyline**	-	-
	**Divalproex**	2.63 (0.91, 8.79)	0.62 (0.12, 3.11)	1.58 (0.18, 14.74)	1.67 (0.26, 9.65)	-	**Divalproex**	0.24 (−2.21, 2.67)
	**Valproate**	**11.38 (1.31, 111.11)**	2.66 (0.22, 32.35)	7.00 (0.37, 128.51)	7.19 (0.51, 94.89)	-	4.30 (0.38, 52.31)	**Valproate**
All Adverse Events								
Nausea	**Placebo**	**Placebo**	**2.44 (1.55, 3.88)**	1.09 (0.47, 2.54)	1.66 (0.70, 4.01)	**4.66 (1.74, 12.93)**	1.13 (0.51, 2.57)	-
	**Topiramate**	1.37 (0.99, 1.94)	**Topiramate**	0.45 (0.18, 1.07)	0.69 (0.25, 1.89)	1.92 (0.72, 5.20)	0.46 (0.19, 1.15)	-
	**Propranolol**	1.13 (0.56, 2.24)	0.81 (0.44, 1.59)	**Propranolol**	1.53 (0.45, 5.26)	**4.29 (1.24, 15.39)**	1.04 (0.39, 2.75)	-
	**Gabapentin**	0.91 (0.47, 1.86)	0.67 (0.32, 1.43)	0.82 (0.32, 2.06)	**Gabapentin**	2.80 (0.75, 10.80)	0.68 (0.20, 2.19)	-
	**Amitriptyline**	0.80 (0.32, 1.84)	0.58 (0.24, 1.37)	0.71 (0.23, 1.89)	0.85 (0.27, 2.50)	**Amitriptyline**	**0.24 (0.07, 0.85)**	-
	**Divalproex**	**3.04 (1.72, 6.47)**	**2.24 (1.13, 4.93)**	**2.79 (1.19, 6.48)**	**3.31 (1.31, 9.25)**	**3.83 (1.40, 12.81)**	**Divalproex**	-
	**Valproate**	2.05 (0.75, 5.65)	1.53 (0.54, 4.07)	1.88 (0.53, 5.59)	2.27 (0.55, 7.43)	2.63 (0.63, 10.29)	0.67 (0.20, 2.32)	**Valproate**
Somnolence								
Dizziness	**Placebo**	**Placebo**	1.36 (0.48, 3.76)	2.68 (0.39, 21.57)	2.68 (0.55, 14.14)	2.20 (0.20, 23.60)	2.95 (0.55, 13.06)	2.16 (0.25, 17.88)
	**Topiramate**	1.17 (0.48, 2.71)	**Topiramate**	1.98 (0.31, 15.33)	1.96 (0.32, 14.39)	1.58 (0.19, 14.47)	2.18 (0.31, 13.03)	1.56 (0.24, 10.53)
	**Propranolol**	1.40 (0.11, 17.00)	1.20 (0.09, 17.20)	**Propranolol**	1.01 (0.07, 12.38)	0.81 (0.04, 13.07)	1.09 (0.07, 10.90)	0.80 (0.04, 10.81)
	**Gabapentin**	**3.69 (1.17, 9.63)**	3.21 (0.74, 11.22)	2.64 (0.19, 35.35)	**Gabapentin**	0.83 (0.04, 12.96)	1.12 (0.10, 8.96)	0.80 (0.05, 11.62)
	**Amitriptyline**	1.63 (0.46, 6.07)	1.40 (0.44, 5.06)	1.17 (0.08, 18.83)	0.44 (0.10, 2.67)	**Amitriptyline**	1.43 (0.07, 20.34)	0.97 (0.05, 15.68)
	**Divalproex**	1.37 (0.05, 34.88)	1.18 (0.04, 35.19)	0.94 (0.11, 9.66)	0.38 (0.01, 11.64)	0.84 (0.02, 27.74)	**Divalproex**	0.73 (0.06, 10.84)
	**Valproate**	0.44 (0.04, 2.98)	0.37 (0.03, 3.27)	0.30 (0.01, 7.28)	0.12 (0.01, 1.13)	0.26 (0.02, 2.72)	0.29 (0.01, 15.46)	**Valproate**
Withdrawal								
Withdrawal due to AEs	**Placebo**	**Placebo**	1.10 (0.95, 1.38)	0.81 (0.63, 1.29)	1.17 (0.80, 2.08)	1.19 (0.68, 2.14)	**1.68 (1.14, 3.67)**	1.29 (0.48, 2.68)
	**Topiramate**	**2.33 (1.55, 3.45)**	**Topiramate**	0.71 (0.54, 1.15)	1.04 (0.67, 1.87)	1.09 (0.60, 1.82)	1.48 (0.96, 3.36)	1.14 (0.42, 2.46)
	**Propranolol**	1.51 (0.78, 2.94)	0.64 (0.32, 1.34)	**Propranolol**	1.54 (0.78, 2.63)	1.56 (0.68, 2.56)	**2.09 (1.11, 4.52)**	1.51 (0.49, 3.69)
	**Gabapentin**	1.81 (0.71, 4.58)	0.78 (0.28, 2.18)	1.21 (0.38, 3.72)	**Gabapentin**	1.03 (0.43, 1.95)	1.34 (0.74, 3.37)	1.02 (0.33, 2.38)
	**Amitriptyline**	2.69 (0.93, 7.57)	1.16 (0.43, 3.12)	1.80 (0.51, 5.84)	1.49 (0.35, 5.96)	**Amitriptyline**	1.32 (0.74, 3.73)	1.08 (0.35, 2.33)
	**Divalproex**	**2.25 (1.01, 5.49)**	0.96 (0.41, 2.57)	1.50 (0.57, 4.26)	1.25 (0.38, 4.49)	0.83 (0.24, 3.40)	**Divalproex**	0.68 (0.22, 1.83)
	**Valproate**	2.20 (0.68, 6.92)	0.96 (0.30, 2.97)	1.50 (0.39, 5.28)	1.25 (0.26, 5.38)	0.85 (0.17, 3.58)	0.99 (0.23, 4.28)	**Valproate**

Row treatments were compared to column treatments in the lower diagonal whereas column treatments were compared to row treatments in the upper diagonal

Boldface means significance

**Fig. 6:**
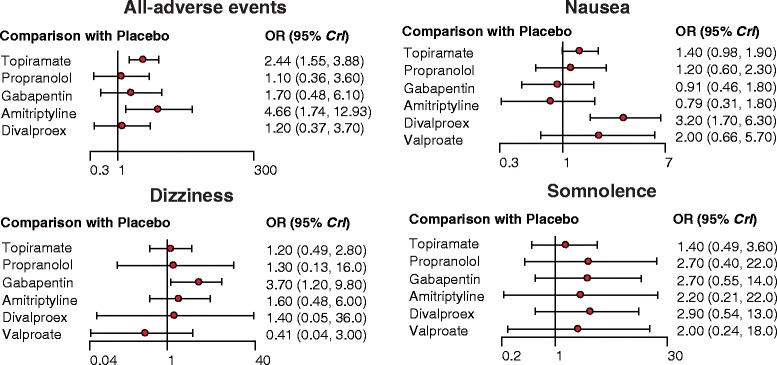
Forest plots of summary effects (NMA) with respect to all adverse events, nausea, dizziness and somnolence

**Fig. 7:**
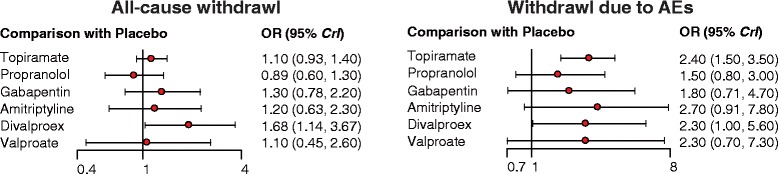
Forest plots of summary effects (NMA) with respect to all-cause withdrawal and withdrawal due to AEs

Our NMA also provides results for the relative safety of migraine interventions. Patients with Divalproex exhibited significantly higher risk of nausea compared to those with placebo, topiramate, propranolol, gabapentin and amitriptyline (all ORs > 1). Furthermore, patients with amitriptyline exhibited a significantly elevated risk of all-adverse events compared to those with propranolol or placebo (all ORs > 1). However, patients with amitriptyline exhibited a significantly reduced risk of all-adverse events compared to those with divalproex (OR = 0.24, 95% CrI = 0.07 to 0.85). Patients with topiramate also exhibited significantly higher risk of all adverse events compared to those with placebo (OR = 2.44, 95% CrI = 1.55 to 3.88; Table 3, Fig. 6). Our NMA also discovered that patients with GABAPENTIN were associated with a significantly increase in the risk of dizziness in comparison to those received placebo (OR = 3.69, 95% CrI = 1.17 to 9.63; Table 3, Fig. 6). The relative tolerability of various migraine interventions were assessed by using the endpoints of all-cause withdrawal and withdrawal due to adverse events. As suggested by Table 3 and Fig. 7, patients with divalproex exhibited a significantly increased risk of all-case withdrawal compared to those with propranolol or placebo (propranolol: OR = 2.09, 95% CrI = 1.11 to 4.52; placebo: OR = 1.68, 95% CrI = 1.14 to 3.67). On the other hand, patients treated with topiramate or divalproex were associated with an increased risk of withdrawals due to adverse events compared to those with placebo (topiramate: OR = 2.33, 95% CrI = 1.55 to 3.45; divalproex: OR = 2.25, 95% CrI = 1.01 to 5.49).

### Ranking of migraine interventions using SUCRA values

**Fig. 8:**
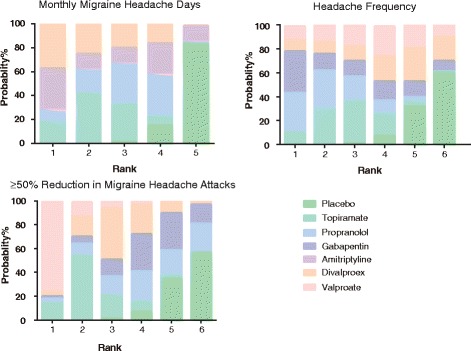
Probability ranking plot of monthly migraine headache days, headache frequency and at least 50% reduction in migraine attacks

**Fig. 9:**
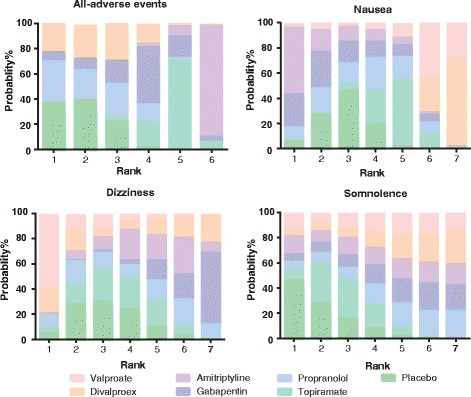
Probability ranking plot of all adverse events, nausea, dizziness and somnolence

**Fig. 10:**
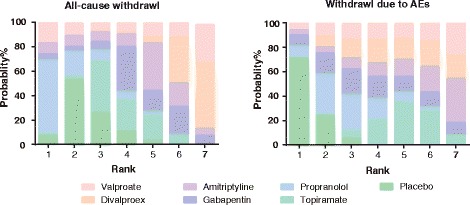
Probability ranking plot of all-cause withdrawal and withdrawal due to AEs

**Fig. 11:**
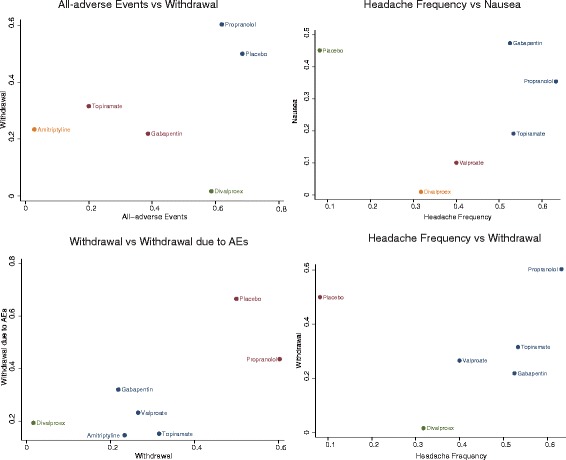
Clustering of migraine interventions. Interventions with the same level of efficacy, safety or tolerability are shown in the same color. Interventions located in the top right corner were more preferable than those in the lower left corner

A ranking plot with respect to each endpoint was produced and a SUCRA table was created in order to differentiate the above migraine interventions (Figs. 8, 9 and 10, Additional file 2: Table S2). Divalproex appeared to have the largest SUCRA value with respect to migraine headache days. Propranolol, topiramate and gabapentin exhibited the largest three SUCRA values with respect to headache frequency. Moreover, valproate, topiramate and divalproex were more preferable than other interventions with respect to the endpoint of at least 50% reduction in migraine attacks. A similar ranking scheme was produced for the above interventions with respect to their safety and tolerability (Additional file 2: Table S2). We also conducted a cluster analysis for grouping the above prophylactic migraine interventions based on the SUCRA values of two endpoints. Overall, propranolol seemed to be the most desirable intervention when several endpoints were simultaneously considered (Fig. 11).

### Assessing consistency between direct and indirect evidence

**Fig. 12:**
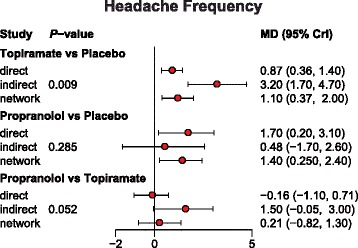
Node-splitting method for assessing consistency with respect to headache frequency

**Fig. 13:**
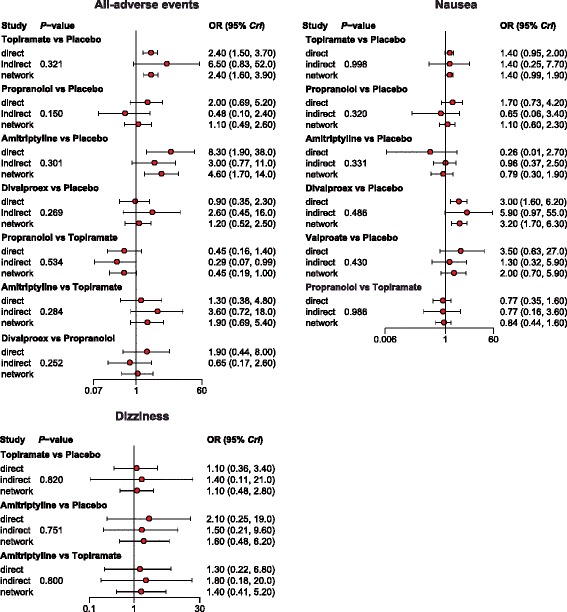
Node-splitting method for assessing consistency with respect to all adverse events, nausea and dizziness

**Fig. 14:**
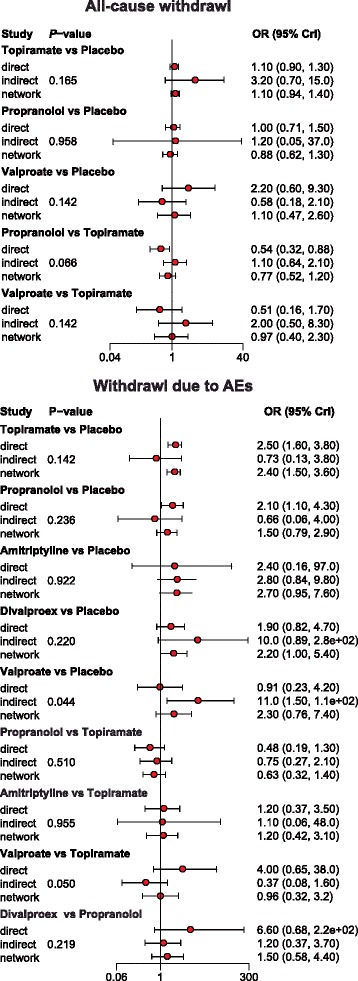
Node-splitting method for assessing consistency with respect to all-cause withdrawal and withdrawal due to AEs

**Fig. 15:**
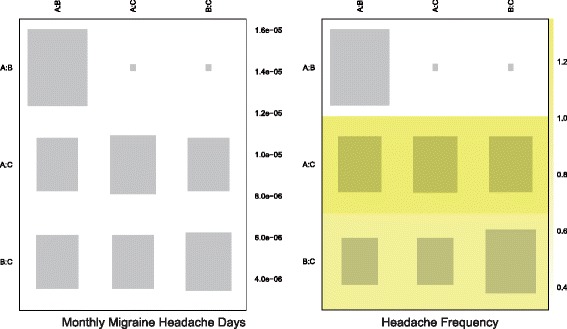
Net heat plot of study designs with respect to monthly migraine headache days and headache frequency. The area of the gray squares displays the contribution of the direct estimate in design d (shown in the column) to the network estimate in design d (shown in the row). The colors are associated with the change in inconsistency between direct and indirect evidence (shown in the row) after detaching the effect (shown in the column). *Blue colors* indicate an increase and warm colors indicate a decrease (the stronger the intensity of the color, the stronger the change)

**Fig. 16:**
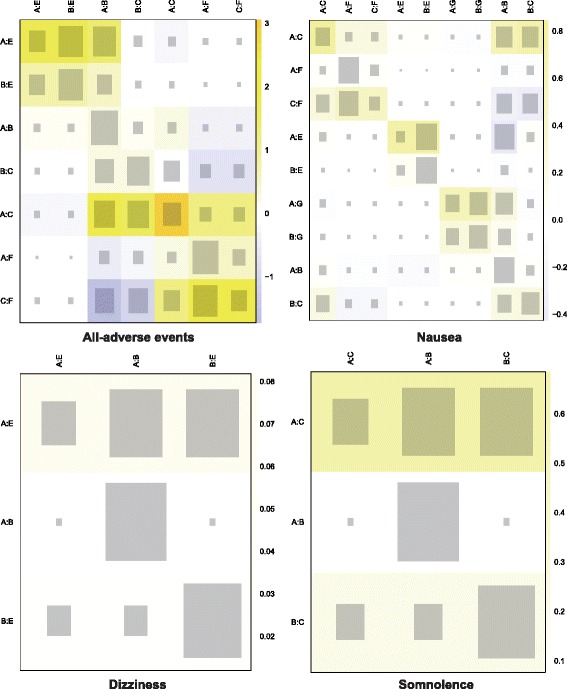
Net heat plot of study designs with respect to all adverse events, nausea, dizziness and somnolence

**Fig. 17:**
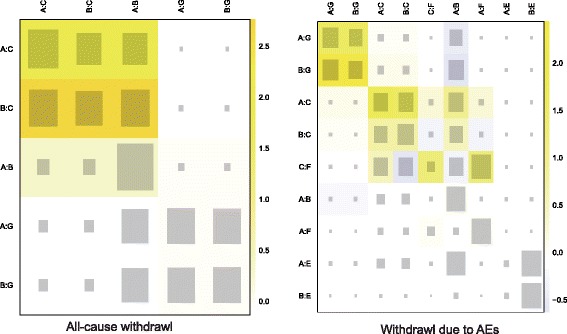
Net heat plot of study designs with respect to all-cause withdrawal and withdrawal due to AEs

Since we implemented a consistency model when conducting the NMA, the node-splitting method was used to assess the validity of this assumption (Figs. 12, 13 and 14). Net heat plots were also produced by software in order to visualize the consistency pattern existed in each comparison (Figs. 15, 16 and 17). As suggested by both the node splitting method (*P*-value > 0.05) and net heat plots, there is no significant inconsistency between direct and indirect evidence for the majority of comparisons. Therefore, we concluded that the consistency model is valid in our NMA.

### Assessing publication bias using funnel plots

Potential publication bias contained in our NMA was evaluated by using the funnel plots produced by software (Additional file 3: Figures S1, Additional file 4: Figure S2 and Additional file 5: Figure S3). As suggested by the funnel plots, there is no significant asymmetry pattern and most studies were evenly distributed in the funnel plot. Therefore, we concluded that there is no significant publication bias in our study.

## Discussion

Migraine is a chronic disabling disease accompanied with recurrent headache. Patients with migraine often suffer from throbbing headache and preventative therapies have been introduced to reduce the risk of migraine onset. Several medications have been applied to migraine patients as prophylaxis and most of these medications are able to reduce the monthly attack frequency by 50% [46]. We conducted an extensive literature review and NMA in order to determine the relative efficacy, safety and tolerability of six popular prophylactic migraine interventions: topiramate, propranolol, gabapentin, amitriptyline, divalproex and valproate.

Results of our NMA indicated that three interventions may be particularly efficacious for reducing the corresponding symptoms of migraine: divalproex, propranolol and valproate. In our study, divalproex ranked the highest with respect to the reduction of monthly headache days whereas propranolol appeared to be the most preferable intervention for reducing headache frequency. Moreover, our study also suggested that valproate exhibited superior performance with respect to at least 50% reduction in headache attacks. Accordingly to the American Academy of Neurology (AAN) and the American Society of Headache (AHS), divalproex is classified as level-A medication and it is offered to patients for migraine prophylaxis [47]. Another study conducted by Kaniecki et al. revealed that both divalproex and propranolol significantly reduced headache frequency and the number of headache days compared to placebo, however, there was no significant difference in the efficacy between the two interventions [48]. The above conclusions were verified by our NMA which did not suggest any significant difference in the efficacy between divalproex and propranolol. As suggested by AAN and AHS, valproate is also classified as level-A medication that should be offered to migraine patients [49]. The efficacy of valproate in reducing migraine attacks has been verified by several studies, for instance, Sørensen et al. was the first one who suggested that valproate exhibited a noteworthy effect on patients with severe migraine with respect to migraine prophylaxis [50]. Although our study suggested that patients with valproate were more likely to experience at least 50% reduction in migraine attacks than those with placebo, the wide confidence interval resulted from potential inconsistency or inadequate evidence should be addressed by conducting large-scale studies in order to verify the above conclusions.

Apart from efficacy, the safety of migraine medication is another predominating factor that must be considered by physicians when selecting an appropriate intervention. As suggested by previous studies, migraine patients treated by antiepileptic drugs may experience several side-effects, including nausea, dizziness and paresthesia [51]. One significant result produced by our NMA is that patients with divalproex exhibited a significantly increased risk of nausea compared to those with placebo or other interventions. This result was confirmed by our SUCRA ranking tables in which the SUCRA value of divalproex appeared to be the lowest with respect to the endpoint of nausea. Apart from that, our pairwise meta-analysis discovered that patients with divalproex were associated with a significantly increased risk of nausea compared to those with placebo. Furthermore, our NMA revealed that patients with divalproex may have poor medication compliance since they appeared to have an increased risk of withdrawal. We urged future researchers to design and conducted prospective studies in order to confirm the safety of divalproex.

Despite that some new findings have been suggested by our study, it is essential to discuss several key issues that may have impact on our conclusions. Firstly, we include both RCTs and crossover studies in our NMA; this may significantly increase the heterogeneity resulted from the design and implantation of different studies. For instance, crossover studies involves randomly assigning a sequence of interventions to different groups over the study period, therefore, the randomization technique used in crossover studies was completely different from that in RCTs. However, the inclusion of crossover studies did not enable us to adjust for the corresponding sequences where a serious of treatments was assigned. Furthermore, the inclusion of crossover studies produced some extra confounding factors that were not presented in RCTs. For instance, a wash-out period between interventions is often used in crossover studies and the duration of the wash-out period may have significant impact on medication compliance as well as on the corresponding endpoints. Our NMA did not adjust for the corresponding dose used for each intervention either. The above uncontrolled factors may independently affect our conclusion or interacted with each other, producing significant effect modification. Nevertheless, the corresponding conclusions and limitations underlying our study provide researchers with key guidelines for designing new trials or prospective studies for migraine patients.

## Conclusio﻿ns

Our NMA suggested that topiramate, propranolol and divalproex may be more efficacious than other prophylactic medications. However, based on the above limitations, our results need to be interpreted with caution. Besides, safety and tolerability of divalproex should be further verified by future studies.

## Additional files

Additional file 1:Table S1. Jadad scale of 32 studies included. (DOCX 15 kb)
Additional file 2: Table S2. Ranking of migraine interventions using SUCRA values. (DOCX 17 kb)
Additional file 3: Figure S1. Funnel plot of studies assessing the efficacy of migraine interventions. (EPS 1495 kb)
Additional file 4: Figure S2. Funnel plot of studies assessing the safety of migraine interventions. (EPS 1629 kb)
Additional file 5: Figure S3. Funnel plot of studies assessing the tolerability of migraine interventions. (EPS 1394 kb)
