# Supplementary material for: Unveiling the relative efficacy, safety and tolerability of prophylactic medications for migraine: pairwise and network-meta analysis
Source: J Headache Pain. 2017 Feb 20;18(1):26. doi: 10.1186/s10194-017-0720-7 (PMC5318356; doi:10.1186/s10194-017-0720-7)
Supplement: Additional file 1:Table S1. — Jadad scale of 32 studies included. (DOCX 15 kb) [file 10194_2017_720_MOESM1_ESM.docx]

**Table S1. Jadad scale of 32 studies included**

| **Author, Year** | **Design** | **Blinding** | **All-cause discontinued** |
| --- | --- | --- | --- |
| Silberstein, 2013 | 2 | 2 | 1 |
| Afshari, 2012 | 2 | 2 | 1 |
| Lipton, 2011 | 2 | 2 | 1 |
| Holroyd, 2010 | 2 | 2 | 1 |
| Dodick, 2009 | 2 | 2 | 1 |
| Ashtari, 2008 | 2 | 2 | 1 |
| Silberstein, 2007 | 2 | 2 | 1 |
| Gupta, 2007 | 0 | 2 | 1 |
| Diener, 2007 | 2 | 2 | 1 |
| Diener, 2007 | 2 | 2 | 1 |
| Tommaso, 2007 | 2 | 2 | 1 |
| Silberstein, 2006 | 2 | 2 | 0 |
| Shaygannejad, 2006 | 0 | 2 | 0 |
| Brandes, 2006 | 2 | 2 | 1 |
| Silberstein, 2004 | 2 | 2 | 1 |
| Mei, 2004 | 2 | 2 | 1 |
| Diener, 2004 | 2 | 2 | 1 |
| Brandes, 2004 | 2 | 2 | 1 |
| Freitag, 2002 | 2 | 2 | 1 |
| Storey, 2001 | 2 | 2 | 1 |
| Mathew, 2001 | 2 | 2 | 1 |
| Klapper, 1997 | 2 | 1 | 1 |
| Kaniecki , 1997 | 2 | 1 | 1 |
| Diener, 1996 | 2 | 2 | 1 |
| Bendtsen, 1996 | 0 | 2 | 0 |
| Mathew, 1995 | 2 | 2 | 1 |
| Hering, 1992 | 0 | 2 | 1 |
| Pradalier, 1989 | 2 | 2 | 1 |
| Mikkelsen, 1986 | 0 | 2 | 1 |
| Sadeghian, 2015 | 2 | 2 | 1 |
| Sarchielli, 2014 | 2 | 2 | 1 |
| Nofal, 2014 | 2 | 2 | 0 |
