# Supplementary material for: Unveiling the relative efficacy, safety and tolerability of prophylactic medications for migraine: pairwise and network-meta analysis
Source: J Headache Pain. 2017 Feb 20;18(1):26. doi: 10.1186/s10194-017-0720-7 (PMC5318356; doi:10.1186/s10194-017-0720-7)
Supplement: Additional file 2: Table S2. — Ranking of migraine interventions using SUCRA values. (DOCX 17 kb) [file 10194_2017_720_MOESM2_ESM.docx]

**Table S2 Ranking of migraine interventions using SUCRA values**

| **Treatment** | **Migraine Headache Days** | **Headache Frequency** | **≥50% Reduction** | **All-adverse Events** | **Nausea** | **Somnolence** | **Dizziness** | **Withdrawal** | **Withdrawal due to AEs** |
| --- | --- | --- | --- | --- | --- | --- | --- | --- | --- |
| Placebo | 0.034 | 0.082 | 0.087 | 0.685 | 0.451 | 0.583 | 0.407 | 0.500 | 0.666 |
| Topiramate | 0.540 | 0.533 | 0.612 | 0.200 | 0.191 | 0.439 | 0.333 | 0.316 | 0.153 |
| Propranolol | 0.406 | 0.632 | 0.298 | 0.620 | 0.354 | 0.206 | 0.281 | 0.603 | 0.437 |
| Gabapentin | - | 0.525 | 0.282 | 0.387 | 0.474 | 0.201 | 0.049 | 0.219 | 0.321 |
| Amitriptyline | 0.462 | - | - | 0.028 | 0.561 | 0.274 | 0.194 | 0.234 | 0.147 |
| Divalproex | 0.566 | 0.318 | 0.460 | 0.587 | 0.009 | 0.174 | 0.306 | 0.017 | 0.194 |
| Valproate | - | 0.400 | 0.755 | - | 0.100 | 0.276 | 0.570 | 0.266 | 0.233 |

AEs: adverse events
